# Sulforaphane synergistically enhances the cytotoxicity of arsenic trioxide in multiple myeloma cells via stress-mediated pathways

**DOI:** 10.3892/or.2012.1977

**Published:** 2012-08-22

**Authors:** NICOLE A. DOUDICAN, SHIH YA WEN, AMITABHA MAZUMDER, SETH J. ORLOW

**Affiliations:** 1 The Ronald O. Perelman Department of Dermatology, New York University School of Medicine, New York, NY, USA; 2 Department of Cell Biology, New York University School of Medicine, New York, NY, USA; 3 New York University Cancer Institute, New York, NY, USA

**Keywords:** sulforaphane, arsenic trioxide, unfolded protein response, Iκβ, endoplasmic reticulum stress, reactive oxygen species

## Abstract

Persistent paraprotein production in plasma cells necessitates a highly developed rough endoplasmic reticulum (ER) that is unusually susceptible to perturbations in protein synthesis. This biology is believed to account for the exquisite sensitivity of multiple myeloma (MM) to the proteasomal inhibitor bortezomib (BTZ). Despite remarkable response rates to BTZ in MM, BTZ carries the potential for serious side-effects and development of resistance. We, therefore, sought to identify therapeutic combinations that effectively disrupt proteostasis in order to provide new potential treatments for MM. We found that sulforaphane, a dietary isothiocyanate found in cruciferous vegetables, inhibits TNFα-induced Iκβ proteasomal degradation in a manner similar to BTZ. Like BTZ, sulforaphane synergistically enhances the cytotoxicity of arsenic trioxide (ATO), an agent with clinical activity in MM. ATO and sulforaphane co-treatment augmented apoptotic induction as demonstrated by cleavage of caspase-3, -4 and PARP. The enhanced apoptotic response was dependent upon production of reactive oxygen species (ROS) as demonstrated by glutathione depletion and partial inhibition of the apoptotic cascade after pretreatment with the radical scavenger N-acetyl-cysteine (NAC). Combination treatment resulted in enhanced ER stress signaling and activation of the unfolded protein response (UPR), indicative of perturbation of proteostasis. Specifically, combination treatment caused elevated expression of the molecular chaperone HSP90 (heat shock protein 90) along with increased PERK (protein kinase RNA-like endoplasmic reticulum kinase) and eIF2α phosphorylation and XBP1 (X-box binding protein 1) splicing, key indicators of UPR activation. Moreover, increased splicing of XBP1 was apparent upon combination treatment compared to treatment with either agent alone. Sulforaphane in combination with ATO effectively disrupts protein homeostasis through ROS generation and induction of ER stress to culminate in inhibition of protein secretion and apoptotic induction in MM. Our results suggest that sulforaphane deserves further investigation in combination with ATO in the treatment of MM.

## Introduction

Multiple myeloma (MM) is a malignancy of terminally differentiated B cells accounting for ~10% of all hematological malignancies and affecting >20,000 patients each year in the United States (1). Despite recent advances in targeted therapies and regimens of high dose chemotherapy with autologous stem cell transplant, there is still no curative treatment. Relapse of disease and development of resistance are major obstacles to overcome for improving treatment response and patient survival in MM (2).

An expansive, highly developed rough endoplasmic reticulum (ER) specialized for constant synthesis and secretion of large amounts of immunoglobulin protein is a defining characteristic of plasma cells. The innate biology of this class of cells renders MM exquisitely sensitive to agents like the proteasome inhibitor bortezomib (BTZ). By virtue of its proteasomal inhibitory activity, BTZ causes accumulation of misfolded proteins in the endoplasmic reticulum (ER), with the resultant ER stress triggering activation of the unfolded protein response (UPR) and apoptosis (3). BTZ has demonstrated remarkable response rates in both relapsed and newly diagnosed MM, but it carries the potential for development of resistance and serious side effects. For example, >30% of patients receiving BTZ treatment develop painful peripheral neuropathy (4).

ER stress triggers the unfolded protein response (UPR), a cellular process activated when unfolded or misfolded proteins accumulate in the lumen of the ER. In this capacity, the UPR’s primary purpose is to restore normal cellular function by halting protein translation and activating signaling pathways to increase the production of molecular chaperones like HSP90 involved in protein folding. If proteostasis is not restored in a timely fashion, the aim of the UPR shifts to promote apoptosis (5). Key mediators of this process include PERK (protein kinase RNA-like ER kinase), eIF2α (eukaryotic translation initiation factor 2-α), XBP1 (X-box binding protein 1) and CHOP (CCAAT/-enhancer-binding protein homologous protein). Upon initiation of UPR activation, PERK undergoes phosphorylation and oligomerization to cause translational attenuation by directly phosphorylating the α subunit of the regulating initiator of the mRNA translation machinery, eIF2 (6). Simultaneously, in a parallel arm of UPR pathway activation, the mRNA of transcription factor XBP1 is spliced. In its activated form XBP1 mRNA encodes for a transcription factor that targets and induces expression of genes containing an unfolded protein response element (UPRE). These genes include ER chaperones, heat shock proteins and XBP1 itself (7). Another effector of the UPR is the transcription factor CHOP. Subsequent upregulation of certain CHOP target genes promotes induction of ER-stress mediated apoptosis (8).

Given its remarkable rates of induction of remission and enhanced long-term survival in the treatment of acute promyelocytic leukemia (9), the utility of arsenic trioxide (ATO) (10) in the treatment of MM has recently been evaluated. *In vitro* models as well as preclinical studies suggest that ATO is able to induce apoptosis at clinically achievable concentrations in drug-resistant MM cell lines and is well tolerated (11,12). Since then, a number of clinical trials have provided evidence for the efficacy of ATO in the treatment of relapsed or refractory MM patients. However, like most drugs used in the treatment of MM, >50% of patients with refractory or relapsed disease eventually present with resistance to ATO when it is used as a single agent (13). In addition to other reported mechanisms of action, ATO has been shown to disrupt calcium stores and promote ER stress-related signaling (14–16).

Sulforaphane (4-methylsulfinylbutyl isothiocyanate), erysolin (4-methylsulfonylbutyl isothiocyanate) and erucin (4-methythiobutyl isothiocyanate) are naturally occurring isothiocyanates that account for the chemopreventative effects of cruciferous vegetables such as broccoli and Brussels sprouts. Sulforaphane is a well characterized inducer of several phase II detoxification enzymes including glutathione-S-transferases and quinone reductase (17). In addition to its chemopreventative effects, sulforaphane has also been reported to cause growth inhibition and induction of apoptosis in a variety of human cancer cell lines (18,19). More recently, proteasomal inhibitory activity has been attributed to the isothiocyanates (20,21).

We have previously shown that ATO and sulforaphane synergize to induce apoptosis in leukemic cells via a reactive oxygen species (ROS)-dependent mechanism (22). Given previously published studies demonstrating the synergistic relationship between ATO and BTZ as well as sulforaphane’s purported proteasomal inhibitory activity (21,23), we wanted to examine the efficacy of sulforaphane in combination with ATO in MM cells. Our ultimate goal is to identify effective combinations that could provide the clinical benefit of BTZ by targeting similar pathways while minimizing debilitating side effects or the emergence of resistance. Here, we report that isothiocyanates block TNFα induced degradation of Iκβ in MM cells in a manner similar to BTZ. Because this inhibition is consistent with reported proteasomal inhibition by isothiocyanates, we investigated potential synergy with ATO. We show that sulforaphane synergizes with ATO in a panel of MM cell lines. Combination treatment results in generation of ROS and ER stress, culminating in enhanced UPR signaling which induces apoptosis.

## Materials and methods

### Cell culture and reagents

Human multiple myeloma cell lines were cultured in Dulbecco’s modified Eagle’s medium (DMEM) supplemented with 10% fetal bovine serum, 2 mmol/l L-glutamine, 5 U/ml penicillin, and 5 μg/ml streptomycin. PCNY-1, MM1.S, MM1.R, KMS-11 and ARP-1 cells were kindly provided by Hearn Cho (New York University School of Medicine, New York, NY, USA). All cells were maintained in an incubator with a humidified atmosphere of 5% CO_2_ at 37°C. Sulforaphane, erysolin, erucin, arsenic trioxide and N-acetyl-l-cysteine (NAC) were purchased from Sigma (St. Louis, MO, USA). Bortezomib was acquired from LC Laboratories (Woburn, MA, USA).

### Cell proliferation assay

For dose response assays, 5,000 cells per well were plated in 96-well culture plates. After overnight incubation, the cells were treated with indicated compounds. Following a 72-h incubation period, cellular proliferation was assessed using a tetrazolium dye reduction assay (CellTiter 96 Aqueous Non-Radioactive Cell Proliferation assay; Promega, Madison, WI, USA) carried out according to the manufacturer’s instructions as previously described (24). Absorbance was recorded on a microplate reader at 495 nm. Cellular proliferation was expressed as a percentage of vehicle-treated cells which were defined as 100% viable. Drug interaction was analyzed by Calcusyn software (Biosoft, Cambridge, UK). This software determines the interaction of two drugs through calculations of the combination index (25) based upon the multiple drug effect equation of Chou and Talalay (26,27). Denotation of CI values as follows: >1, antagonism; 1, additivity; <1, synergy. GI_50_ values, which represent the concentration necessary to cause 50% growth inhibition, were calculated from dose-response curves generated from cell proliferation assays.

### Quantification of cellular glutathione levels

Cellular levels of glutathione (GSH) levels were determined using the HT Glutathione Assay kit from Trevigen (Gaithersburg, MD, USA) as previously described (22). Briefly, 24 h after seeding MM cells at a density of 1×10^6^, cells were exposed to indicated compounds for 3 h at 37°C. The cells were collected and washed with PBS. An equal number of cells from each treatment group were aliquoted for use in cellular GSH quantification per the manufacturer’s instructions.

### Protein extraction and western blotting

Cells were harvested in extraction buffer [1% Triton X-100, 50 mmol/l Tris, 2 mmol/l EDTA, 150 mmol/l NaCl (pH 7.5)] containing protease inhibitor cocktail (Roche Applied Science, Indianapolis, IN, USA) and phosphatase inhibitor cocktail (Sigma) after two washes with ice-cold phosphate buffered saline (PBS). The lysates were centrifuged at 10,000 × g at 4°C for 10 min in a microcentrifuge. Protein supernatants were measured with a protein assay kit (Bio-Rad, Hercules, CA, USA). Proteins were separated by 10% sodium dodecyl sulfate polyacrylamide gel electrophoresis (SDS-PAGE) and transferred onto polyvinylidene difluoride membranes (Polyscreen; Perkin-Elmer, Waltham, MA, USA). Antibodies against cleaved forms of poly(ADP-ribose) polymerase (PARP), caspase-3 and -4 were obtained from Cell Signaling Technology (Danvers, MA, USA). Phospho-PERK, phospho-eIF2α, CHOP and HSP90 antibodies were obtained from Santa Cruz Biotechnology (Santa Cruz, CA, USA). Anti-actin (Sigma) was used as a control. Immunoreactive bands were visualized using enhanced chemiluminescence detection reagent (Perkin-Elmer) and X-OMAT processing.

### XBP1 splicing

Cells were treated with indicated concentrations of ATO and/or sulforaphane for 24 h. Total RNA was isolated from lysed cells with the RNeasy Mini kit (Qiagen, Germantown, MD, USA). XBP1 splicing was assessed by semi-quantitative RT-PCR as described previously (28,29). cDNA was produced from total RNA preps using ImProm-II Reverse Transcription system (Promega). Primers spanning the fragment of XBP1 containing the intron targeted by Ire1α were used: 5′-TACGGGAGAAAACTCACGGC-3′ and 5′-GGGTCCAACTTGTCCAGAATGC-3′. The thermal PCR cycling conditions are as follows: 95°C for 5 min, 95°C for 1 min, 58°C for 30 sec, 72°C for 30 sec, and 72°C for 5 min with 35 cycles of amplification. PCR products were digested with Pst1 (New England Biolabs, Ipswich, MA, USA) before being separated on a 2.0% agarose/1X TAE gel and visualized by ethidium bromide.

### Gaussia luciferase secretion assay

Commercially available lentiviral particles obtained from GenTarget (San Diego, CA, USA) expressing *Gaussia* luciferase (Gluc) were introduced into KMS-11 and ARP-1 MM cells by infection. For infection, cells were cultured in 6-well tissue culture plates to a density of 3×10^6^ cells/ml and then diluted to 1×10^6^ cells/ml in complete media. Lentiviral particle was added at a ratio of 100 μl viral particles to 1 ml of cells. After 24 h, equal parts of fresh media containing puromycin selection were added. After 72 h, efficacy of transduction was assessed by RFP fluorescence. After infection, cells were maintained in media containing 3 μg/ml puromycin to establish stable clones. For secretion assays, 100,000 cells/well were plated in 96-well culture plates. Cells were immediately treated with indicated concentrations of compounds. Following 24-h incubation, expression and secretion of Gluc was monitored using The BioLux *Gaussia* Luciferase Assay kit (New England Biolabs) according to the manufacturer’s instructions through measurements of luciferase activity as indicated by relative light units (RLU) on a microplate luminometer (Molecular Devices, Sunnyvale, CA, USA). Percent reduction in Gluc secretion was determined by the following equation: (RLU of treated cells/RLU ratio of the untreated, DMSO control cells) × 100.

### Statistical analyses

Unless otherwise noted, experiments were performed in triplicate. The data are presented as the average ± SEM. P-values were determined by a two-sided Student’s t-test with unequal variance, with P<0.05 considered significant.

## Results

### Bortezomib enhances ATO-mediated growth inhibition

**Figure 1:**
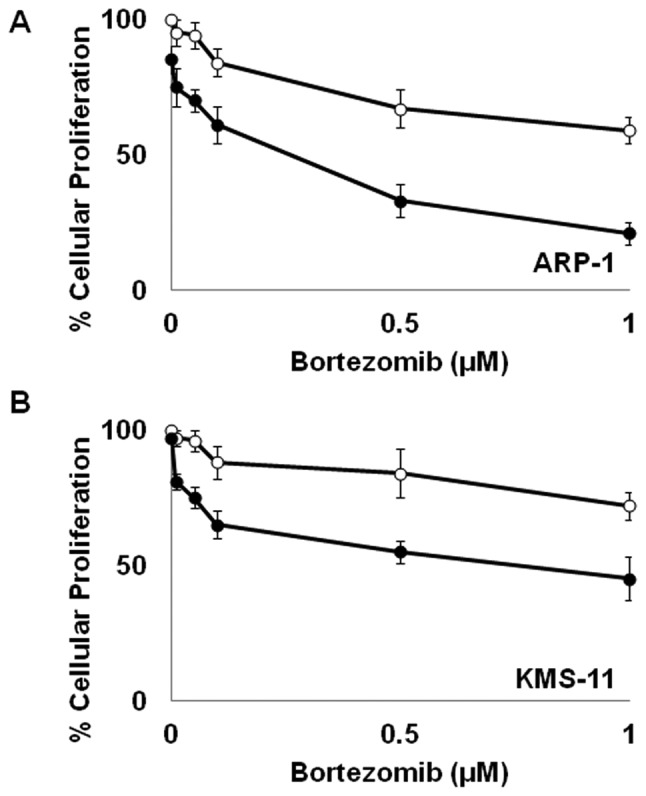
Combination bortezomib and ATO reduce cellular proliferation in MM cells. ARP-1 (A) and KMS-11 (B) cells were treated with indicated concentrations of bortezomib alone (○) or in combination with 0.5 μM ATO (●) for 72 h. All experiments were performed in triplicate with error bars representing SEM.

In order to enhance the potential clinical efficacy of ATO in the treatment of MM, we wanted to assess its interactive potential with BTZ, a current standard of care in the treatment of MM. As shown in Fig. 1, low, subclinical doses of BTZ and ATO as single agents have limited effect on cellular proliferation in MM. However, when used in combination, BTZ and ATO markedly inhibit cellular proliferation in both KMS-11 and ARP-1 cells (Fig. 1).

### Sulforaphane and erysolin inhibit TNFα-induced degradation of Iκβ

**Figure 2:**
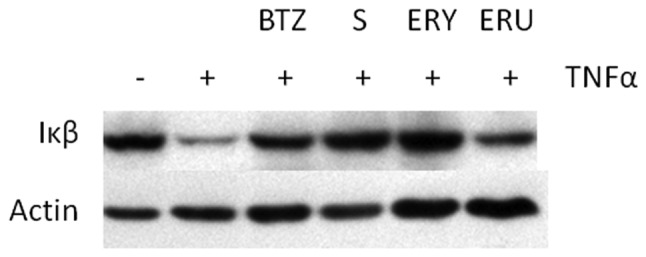
Isothiocyanates inhibit TNFα-induced Iκβ degradation. KMS-11 cells were treated with 20 ng/ml TNFα for 30 min in the presence or absence of 1-h pretreatment with either 10 μM bortezomib (BTZ), 10 μM sulforaphane (S), 10 μM erysolin (ERY) or 10 μM erucin (ERU). Cellular levels of Iκβ were examined by western blot analysis with actin used as a loading control. Blot is representative of 3 independent experiments.

Given that recent studies indicate that isothiocyanates possess anti-proteasomal activity, we hypothesized that isothiocyanates should have biological effects similar to BTZ. In order to test this hypothesis, we examined the effect of isothiocyanates on TNFα induced proteasomal degradation of Iκβ. Upstream activation signaling by TNFα-stimulation causes phosphorylation of Iκβ which is ultimately targeted for degradation by the 26S proteasome (30). As previously reported (31) and shown in Fig. 2, proteasome inhibitors such as BTZ prevent TNFα-induced Iκβ proteasomal degradation. Similarly, sulforaphane and erysolin pretreatment also prevent Iκβ degradation after TNFα stimulation. Erucin pretreatment only minimally inhibits Iκβ degradation.

### Sulforaphane and erysolin enhance ATO growth inhibition in a synergistic fashion

Because these data along with other published studies suggest that isothiocyanates inhibit proteasomal mediated protein degradation, we wanted to investigate their ability to potentiate the growth inhibitory effects of ATO. As shown in Tables I and II, sulforaphane and erysolin display a greater than additive effect (i.e., synergize) with ATO in 3 MM cell lines. Consistent with its inability to inhibit TNFα induced degradation of Iκβ (Fig. 2), erucin interaction with ATO was found to be antagonistic (Table II). Due to its potency, we focused further experiments on sulforaphane.

**Figure 3:**
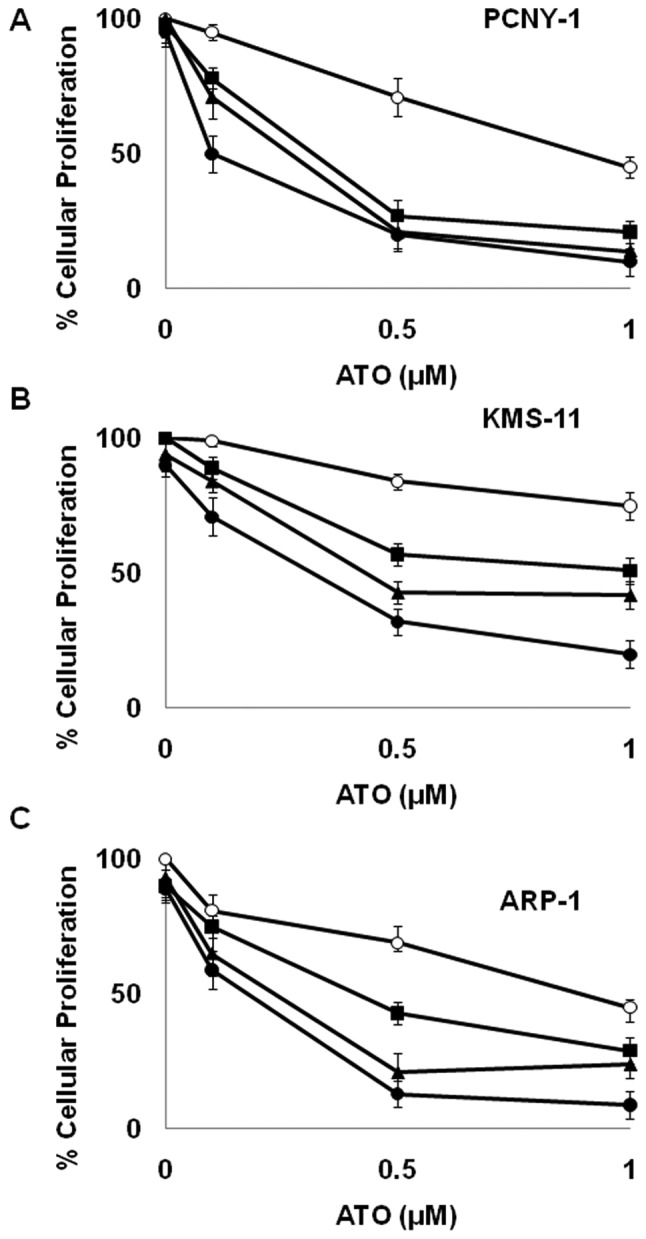
Sulforaphane enhances ATO-mediated growth inhibition in MM cells. PNCY-1 (A), KMS-11 (B) or ARP-1 (C) cells were treated with indicated concentrations of ATO alone (○) or in combination with 1 μM sulforaphane (■), 3 μM sulforaphane (▲), or 5 μM sulforaphane (●) for 72 h. All experiments were performed in triplicate and error bars were calculated using SEM.

As shown in Fig. 3, ATO caused modest growth inhibition in PCNY-1 MM cells when employed as a single agent. Similarly, concentrations up to 5 μM sulforaphane had a minimal effect on the proliferation of MM cells (Fig. 3). However, when used in combination, the ability of these compounds to reduce proliferative capacity was dramatically enhanced. Combination index analysis demonstrates the relationship between 0.5 μM ATO and 3 μM sulforaphane to be strongly synergistic (Table I). Similar effects were observed in 4 out of 5 MM cell lines examined (Fig. 3 and Table I). The synergistic relationship was not observed in MM1.R cells which are a subclone of the MM.1 human MM cell line selected for resistance to glucocorticoid therapy (32).

### Sulforaphane and ATO in combination enhance apoptotic induction through an ROS-dependent mechanism

**Figure 4:**
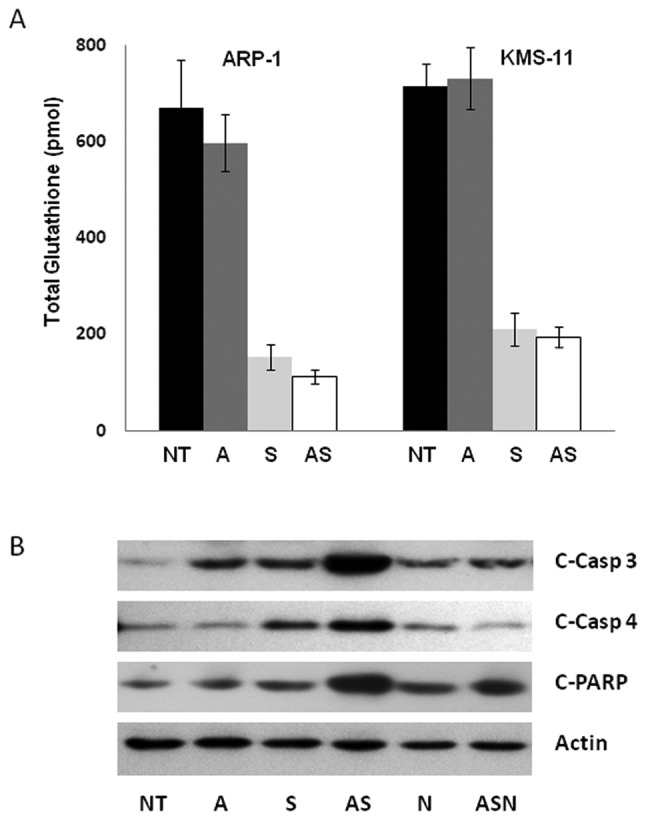
The combination ATO/sulforaphane treatment induces apoptosis partially mediated by ROS generation. (A) KMS-11 cells were incubated with 0.3 μM ATO, 3 μM sulforaphane, or both. After 3 h of treatment, total cellular GSH values were assessed. Experiments were performed in triplicate and error bars were calculated using SEM. (B) KMS-11 cells were incubated with 1 μM ATO, 3 μM sulforaphane, or in combination for 24 h. Proteins were extracted from the cells for analysis by immunoblotting with antibodies to actin, cleaved caspase-3 (c-Casp-3), cleaved caspase-4 (c-Casp 4) and cleaved PARP (c-PARP). Representative blots shown from 3 independent experiments. NT, no treatment; A, ATO; S, sulforaphane; N, NAC.

As we previously demonstrated in leukemic cells (22), sulforaphane also acts as an ATO sensitizing agent through depletion of intracellular GSH in MM cells (Fig. 4A). In this capacity, cellular depletion of glutathione by sulforaphane renders cells largely incapable of protection against ATO’s ability to generate ROS. In order to understand the biological consequences of enhanced ROS, we evaluated the induction of apoptosis via cleavage of effector caspase-3 and poly(ADP-ribose) polymerase (PARP), indicators of apoptosis. As shown in Fig. 4B, higher levels of cleaved caspase-3 and PARP protein were present in KMS-11 cells exposed to the combination treatment of 3 μM sulforaphane and 1 μM ATO than with either agent alone. Similar results were observed in ARP-1 cells (data not shown). These data indicate that combinatorial sulforaphane/ATO treatment enhances the apoptotic response. Interestingly, cleavage of the ER stress specific caspase-4 is also enhanced in response to combination treatment (Fig. 4B).

ROS play a critical role in the apoptotic response of combined ATO and sulforaphane treatment as demonstrated by the fact that preincubation with the ROS scavenger N-acetyl cysteine (NAC) partially attenuated the apoptotic induction of combination treatment (Fig. 4B). Pretreatment with NAC had no effect on apoptosis alone. However, in combination with ATO and sulforaphane, NAC partially inhibited PARP, caspase-3 and -4 cleavage to levels comparable to treatment with ATO alone.

### Combination sulforaphane and ATO treatment promotes ER stress

**Figure 5:**
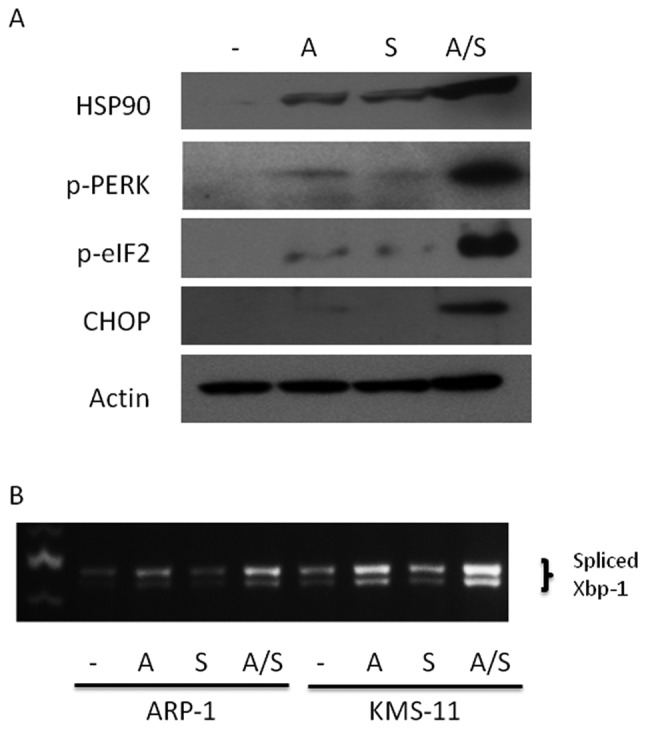
The combination ATO/sulforaphane treatment enhances ER stress and activates the unfolded protein response. (A) KMS-11 cells were incubated with 1 μM ATO, 3 μM sulforaphane or in combination for 24 h. Proteins were extracted from the cells for analysis by immunoblotting with antibodies to HSP90, phospho-PERK (p-PERK), phospho-eIF2α (p-eIF2) and CHOP. Actin was used as a loading control. Blot representative of at least 3 independent experiments. (B) KMS-11 and ARP-1 cells were incubated with 1 μM ATO, 3 μM sulforaphane or in combination for 24 h. After treatment, total RNA was extracted from the cells and *Xbp*1 splicing was assessed by RT-PCR. Image representative of at least 3 independent experiments. NT, no treatment; A, ATO; S, sulforaphane.

Hypothesizing that sulforaphane’s anti-proteasomal activity also is integral to its ability to augment ATO cytotoxicity in MM cells, we investigated whether combination treatment resulted in enhanced ER stress due to perturbations in protein processing. Consistent with this notion, we observed upregulation of HSP90, a general marker for ER stress (33), in KMS-11 cells co-treated with ATO and sulforaphane (Fig. 5A). Additionally, activation of the PERK pathway, a key component of the unfolded protein response (UPR), was enhanced upon co-treatment. As shown in Fig. 5A, PERK phosphorylation was elevated after treatment with ATO and sulforaphane with pathway activation demonstrated by increased expression of downstream mediators CHOP as well as phosphorylation of eIF2. Consistent with upregulation of the PERK arm of the UPR response, parallel activation of the IRE1 arm of the UPR response was observed through enhanced splicing of the UPR transcription factor XBP1 upon treatment with ATO and sulforaphane (Fig. 5B). Similar results were observed in ARP-1 MM cells (data not shown).

### Combination sulforaphane and ATO treatment disrupts protein secretion in MM cells

**Figure 6:**
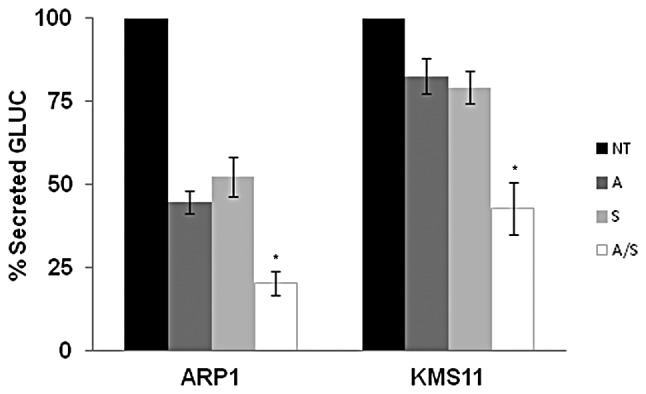
ATO and sulforaphane combination treatment effectively reduces protein secretion. Clones of KMS-11 and ARP-1 cells stably expressing Gluc were treated with 1 μM ATO, 3 μM sulforaphane or in combination for 24 h. After treatment, Gluc secretion was assessed by measuring luciferase activity in the media. Percent secreted Gluc is determined by the following equation: (RLU of treated cells/RLU ratio of the untreated, DMSO control cells) × 100. ^*^P<0.05. All experiments were performed in triplicate and error bars were calculated using SEM. NT, no treatment; A, ATO; S, sulforaphane.

B cells synthesize and secrete immunoglobulin protein. Blocking or decreasing protein processing in the secretory pathway is another hallmark of ER stress that is specifically relevant to the biology of MM cells (34). In order to assess the effects of ATO and sulforaphane on protein secretion, we employed the reporter protein *Gaussia* luciferase (Gluc). Gluc is a naturally secreted luciferase that can be easily monitored through extracellular release of luciferase activity in real time, and has been developed as a sensor of ER stress (35). As shown in Fig. 6, clones of KMS-11 and ARP-1 stably expressing Gluc show a notable reduction in Gluc secretion when treated with 1 μM ATO or 3 μM sulforaphane alone. Consistent with our studies of additional ER stress markers, combination ATO and sulforaphane treatment inhibits protein secretion in a fashion that is greater than treatment with single agent alone. Altogether, these data suggest that when ATO and sulforaphane are administered together, ER stress mediated pathways are enhanced.

## Discussion

In the present study, we demonstrated that the naturally occurring dietary compound sulforaphane inhibited TNFα induced proteasomal degradation of Iκβ in a manner similar to BTZ (Fig. 2). Similarly to BTZ, sulforaphane is a potent ATO sensitizer (Figs. 1 and 3). In 4 out of 5 MM cell lines examined, a synergistic relationship between the compounds was observed when combined with 3 μM sulforaphane (Table I). The synergistic growth inhibition was due to enhanced induction of apoptosis (Fig. 4), in keeping with the the combination’s ability to generate ROS and ER stress, activate UPR signaling and inhibit protein secretion (Figs. 4–6).

The combinatorial effects of ATO and BTZ have been reported in a variety of leukemic cell lines as well as in MM cell lines (25,36). More recently, combination ATO/BTZ regimens have demonstrated synergistic activity against MM in both preclinical and clinical studies (37,38). Indeed, our results show a similar effect of ATO/BTZ in KMS-11 and ARP-1 MM cells (Fig. 1). Previous studies have implicated a variety of mechanisms for the combined anti-proliferative activity of these agents including p38 MAPK activation and proteolytic activation of protein kinase C delta (PKCδ) (23,36). The similarities between ATO/BTZ and ATO/sulforaphane combined growth inhibition along with BTZ and sulforaphane’s described anti-proteasomal activity caused us to hypothesize that induction of ER stress could be implicated as a mechanism of action with the data presented herein supporting that notion. This activity is consistent with the previously described mechanisms of action for BTZ/ATO synergy, as both p38 MAPK and PKCδ are downstream signaling components of ER stress mediated apoptotic pathways (39,40).

We previously demonstrated that ATO and sulforaphane were an effective combination in a panel of non-acute promyelocytic leukemia hematological malignancies (22). In our studies using leukemic cells, we demonstrated that sulforaphane depleted intracellular glutathione levels causing enhanced ROS generation upon combination treatment. Here, we also demonstrated a dependence on ROS for enhanced apoptotic induction in MM cells (Fig. 4), and that combination treatment by sulforaphane and ATO effectively induced ER stress mediated responses such as upregulation of HSP90, activation of the UPR and inhibition of protein secretion (Figs. 5 and 6). Recent studies have suggested that the ER also may play an important role in response to oxidative stress (41,42). Moreover, the ER is exquisitely sensitive to oxidative damage (43). Therefore, given our data suggesting involvement of both ROS and ER stress pathways in response to combination ATO and sulforaphane treatment, we examined any potential interplay between these 2 critical cellular stress responses. Indeed, the antioxidant NAC attenuated ER stress-mediated apoptosis as measured by a reduction in cleavage of the ER specific caspase-4, suggesting ROS involvement in the induction of ER stress specific apoptosis (Fig. 4). Because depletion of glutathione is already observed after only 3 h of treatment, it is possible that ROS act as upstream signaling molecules to initiate UPR pathways and ER stress apoptosis. Nevertheless, further studies to elucidate the specific links between ROS and ER-stress are needed.

Interestingly, the synergistic effects of isothiocyanates with ATO were limited to sulforaphane and erysolin (Table II). The structurally related isothiocyanate erucin did not display synergy with ATO in MM cells (Table II). Moreover, the effect of each isothiocyanate on inhibition of TNFα-induced Iκβ degradation appears to parallel each compound’s combinatorial effect with ATO. For example, both sulforaphane and erysolin synergize with ATO and inhibit Iκβ degradation in a manner similar to BTZ. In contrast, erucin had minimal effect on Iκβ degradation and did not synergize with ATO. Our studies with isothiocyanates in leukemic cells also demonstrated ATO synergism only with sulforaphane and erysolin (22). These data suggest the importance of proteasomal inhibition as a mechanism for disrupting MM cellular proliferation.

One limitation of this combination is the observation that ATO/sulforaphane is not synergistic in MM1.R cells. MM1.R cells are a subclone of the parental MM1 human MM cell line selected for resistance to glucocortocoid therapy through loss of the glucocortocoid receptor. MM1.S cells also used in this study are subclones selected for sensitivity to glucocortocoid therapy. Interestingly, MM1.S displayed a synergism between ATO and sulforaphane, whereas the relationship in MM1.R cells was classified as antagonistic (Table I). According to the literature as well as our own studies, MM1.R cells are not resistant to other proteasome inhibitors like BTZ or carfilzomib (44,45). These data could potentially suggest that the reason for antagonism in MM1.R cells is not based upon a mechanism rooted in ER stress. Interestingly, the glucocorticoid receptor itself has been implicated in NFκβ inactivation through tethering processes which disrupt critical interaction with translational machinery (32). In our study, ATO and sulforaphane are synergistic in MM1.S cells expressing glucocorticoid receptors (GCRs), but antagonistic in MM1.R cells which lack GCRs, which may point to an additional mechanism for regulation of NFκβ in response to cytotoxic stressors. Although not specifically addressed in this study, this is an area of further interest.

Given the clinically validated importance of targeting ER stress pathways in the treatment of MM as exemplified by BTZ (46), our data suggest that the combination ATO and sulforaphane may hold therapeutic potential. It is important to note that BTZ carries the potential for serious side effects with >30% of patients reporting painful peripheral neuropathy (4). In contrast, sulforaphane is a natural product with a well documented safety profile. Moreover, the concentrations used in these studies are clinically achievable after dietary consumption (47). Similarly, the effective concentrations of ATO are clinically relevant (48). Therefore, ATO and sulforaphane combination is deserving of further investigation as a potentially well tolerated yet effective treatment for MM.

## Figures and Tables

**Table I:** Combinatorial effects of ATO and sulforaphane in a panel of MM cell lines.

Cell line	ATO IC_50_ (μM)	ATO + 3 μM sulforaphane IC_50_ (μM)	CI	Interpretation
PCNY-1	0.94	0.26	0.432	Synergistic
KMS-11	1.41	0.54	0.659	Synergistic
ARP-1	1.05	0.33	0.594	Synergistic
MM1.S	0.89	0.49	0.641	Synergistic
MM1.R	2.14	2.02	1.21	Antagonistic

**Table II:** Combinatory effects of isothiocyanates and ATO in a panel of MM cell lines.

Cell line	Combination	CI	Interpretation
PCNY-1	3 μM erysolin, 1 μM ATO	0.646	Synergistic
ARP-1	3 μM erysolin, 1 μM ATO	0.714	Synergistic
KMS-11	3 μM erysolin, 1 μM ATO	0.597	Synergistic
PCNY-1	3 μM erucin, 1 μM ATO	1.02	Antagonistic
ARP-1	3 μM erucin, 1 μM ATO	0.994	Antagonistic
KMS-11	3 μM erucin, 1 μM ATO	1.13	Antagonistic

## Abbreviations

MM: multiple myeloma
ER: endoplasmic reticulum
ROS: reactive oxygen species
BTZ: bortezomib
ATO: arsenic trioxide
CI: combination index
TNFα: tumor necrosis factor alpha
Iκβ: inhibitor of kappa beta
UPR: unfolded protein response
PARP: poly(ADP-ribose) polymerase
PERK: protein kinase RNA-like endoplasmic reticulum kinase
eIF2α: eukaryotic translation initiation factor 2α
XBP1: X-box binding protein 1
NAC: N-acetyl-cysteine
